# The experiences of underrepresented minority faculty in schools of medicine

**DOI:** 10.3402/meo.v19.24768

**Published:** 2014-12-02

**Authors:** Dena Hassouneh, Kristin F. Lutz, Ann K. Beckett, Edward P. Junkins, LaShawn L. Horton

**Affiliations:** 1 School of Nursing, Oregon Health & Science University, Portland, OR, USA; 2 Department of Pediatrics, College of Osteopathic Medicine of the Pacific-Northwest, Western University of Health Sciences, Lebanon, OR, USA

**Keywords:** academic medicine, diversity, faculty of color, grounded theory, recruitment, retention, qualitative research, underrepresented minorities

## Abstract

**Introduction:**

Faculty of color in schools of medicine play an essential role in addressing health disparities, increasing diversity in healthcare, and improving health professions education. Yet inadequate progress has been made in increasing the numbers of faculty of color in medical schools. The reasons for this gap, and ways to address it, are poorly understood.

**Methods:**

We conducted a grounded theory study of 25 of faculty from groups historically underrepresented in academic medicine at 17 schools in the United States. Faculty were interviewed in person (*n*=4, 16%) and by telephone (*n*=21, 84%).

**Results:**

We identified two processes that contribute to a greater understanding of the experiences of faculty of color: patterns of exclusion and control, and surviving and thriving. We also identified one outcome – faculty of color having influence.

**Conclusions:**

Strong support from leaders, mentors, and peers to nurture and protect faculty of color in schools of medicine is needed to counteract the negative effects of racism and to promote the positive effects this group has on diversity and excellence in medical education. Specific strategies for survival and success are described.

Nationally, schools of medicine strive to improve patient outcomes through teaching, research, and practice. Yet substantial segments of the United States population still face significant barriers to health care access and quality (1–3). Evidence suggests that lack of faculty diversity in schools of health education is a factor in perpetuating these disparities (4). Faculty of color are more likely to provide health care in underserved regions than majority faculty. In academic medicine, they serve as important role models and mentors for minority students and trainees and promote academic excellence that improves student outcomes in cultural competence, humanism, and professionalism (1). However, the Association of American Medical Colleges points out that arguing for faculty diversity's dividends is insufficient (1). Diversity must be seen as a driver of excellence in medical education, essential to and congruent with other calls for education reform such as those outlined by the recent Carnegie report on educating physicians (5).

The terms ‘faculty of color’ and ‘underrepresented minority faculty’ (URM) refer to two overlapping but distinct groups. The former includes Asians who are minorities in the US population but not in medicine. The latter are people of African American, Native American, and Latino descent. The medical education literature provides information on both groups. In a comprehensive review of the literature on faculty of color experiences in academe, Turner, Gonzalez, and Wood found that the experiences of faculty of color in general and URM in particular are socially complex and have personal meaning. Thus, qualitative approaches are appropriate for studying factors that affect these groups (6).

Despite the recognized importance of diversity in medical education, we identified only five qualitative studies investigating various aspects of faculty of color or URM experiences in academic medicine (7–11). All were descriptive studies; only two included national samples (7, 10), with only one of the two sampling faculty of color exclusively (7). Common findings in these five studies include the critical importance of mentorship (8–11), the pervasive nature and influence of racism in academe (7, 9–11), and a feeling of isolation among faculty of color (8, 10, 11). This paper focuses on medical education. However, our analysis draws from a larger national study (*n*=95), including nursing, medicine, pharmacy, and dentistry. The aims of this larger study were to: 1) explore the influence of racism on faculty of color in health professions education; 2) identify strategies to support the recruitment, retention, and success of faculty of color in health professions education; and 3) develop a substantive grounded theory of faculty of color experiences in predominantly White health professions schools. In this paper, we describe and provide evidence for the three major components of our theory of how faculty of color survive and thrive in health professions education, using examples of URM medical school faculty. We focus exclusively on URM here to strengthen representation of the voices of this important group in the literature. In this paper, we will use the term ‘faculty of color’ when describing the theory and sample as a whole. We will use ‘URM’ when referring to findings and implications that are specific to URM faculty.

## Review of literature

Faculty of color serve as mentors and role models for students of color, help to dispel myths and stereotypes about people of color, and as a result of their experiences as minorities, bring particularly informed perspectives on social justice to the classroom and faculty service (12). In addition, faculty of color take on greater teaching, mentoring, service, and administrative/committee responsibilities relative to White faculty overall and are more likely to use active pedagogical techniques that improve student outcomes (13). Because they serve as role models and mentors on predominantly White campuses, faculty of color improve the recruitment, retention, and success of junior faculty and students of color, thereby improving campus diversity and supporting pipeline efforts (14). Despite the importance of faculty of color, African Americans, Native Americans, and Latinos are underrepresented in all areas of higher education (13). In medicine, only meager gains have been seen among URM faculty, with increases from 6.8 to 8% between 2000 and 2010 (15). This small increase in URM faculty of 1.2% has not kept pace with the growth in URM medical school graduates. The number of African American, Native American, and Latino graduates combined and averaged as a whole has increased 17.6% between 2002 and 2011 (15). Jayakumar and colleagues point to a similar discrepancy in the poor growth of underrepresented minority faculty in higher education generally and the increasing number of persons of color graduating with doctorates over the past 40 years. They conclude that while pipeline issues remain a concern, addressing institutional racism is critical to improving representation of faculty of color (13).

The higher education literature is replete with evidence that diverse faculty experience disadvantages in predominantly White institutions (6, 16–19). This disadvantage is particularly significant for underrepresented minorities. Faculty who find themselves underrepresented or alone often experience emotional discomfort resulting from heightened visibility, the tendency to judge them as representatives of their race, the effects of ethnic or racial stereotyping, and the experience of social isolation (12). Unequal treatment is also commonly reported (16, 17, 20, 21). Faculty of color receive less support for their teaching and research and experience a more challenging path for promotion and tenure relative to White faculty (13). These disadvantages lead to feelings of dissatisfaction. Allen and colleagues found differences in levels of satisfaction by race. Whereas 37% of White faculty indicated the highest level of satisfaction with their institution, only 23% of African American faculty were equally satisfied (22). African American women reported the greatest dissatisfaction highlighting the intersecting effects of racism and sexism in academe. Specifically, African American women were least likely to report that their general institutional satisfaction was ‘very good’ and reported the highest levels of dissatisfaction with their pay relative to other groups.

Although much information is available documenting the hostile treatment of faculty of color in higher education, less information is available about how to counteract these negative patterns. What is known suggests that supportive administrative leadership, mentorship relationships, networking, and interaction with other faculty of color are all important positive factors related to faculty persistence and success (12, 23–25). Care of the self, using a strengths-based lens to view one’s own career, and staying true to oneself are also important strategies (12, 23, 24, 26, 27). Stanley also suggests learning self-advocacy skills, finding allies, knowing the culture and rules of one's institution, learning to protect one's time, being productive, and being prepared to respond skillfully to racism and other systems of oppression in academe as useful strategies for retention of faculty of color (24).

## Methods

### Design

Grounded theory is a systematic, inductive, and comparative approach for conducting inquiry with the explicit purpose of constructing theory (28). We studied the experiences of faculty of color at predominantly White medical schools using a grounded theory approach situated in the critical paradigm (29–31). Some of the basic assumptions of the critical paradigm include the belief that all thought is fundamentally mediated by power relations that are socially and historically constituted, that certain groups in any society are privileged over others, and that language is central to the formation of subjectivity (32). Thus, a critical grounded theory approach builds on and revises the classical grounded theory of Glaser and Strauss by acknowledging the constructed nature of knowledge and knowledge development, while also recognizing the importance of privileging the standpoint of oppressed groups in the research process (28–34). Although we will present the processes of data collection and analysis in a somewhat linear fashion for clarity and to accord with convention, in grounded theory, these processes are integrated and interdependent.

### Sample

Our medical school faculty sample included 29 self-identified faculty of color who were employed at predominantly White schools of medicine; 25 were URM (see Table 1). To recruit faculty from different academic ranks, geographic locations, races, and ethnicities, we used maximum variation sampling. Our URM SOM sample included faculty from all academic ranks in states from every region in the continental US. Participants worked in public and private colleges and universities, both research-intensive and teaching-focused. We recruited participants through medical educators’ listserv, professional organizations and meetings, personal contacts, and snowball sampling. Recruitment and data collection of the SOM sample occurred in 2012 (Table 2).

**Table 1:** Sample demographics (*N*=29 medicine)

Demographic characteristic		*n* (%)
Race/ethnicity	African American	19 (66)
	Asian American	4 (14)
	Latino	5 (17)
	Native American	1 (3)
Immigrant status	Immigrant	4 (14)
	US born	25 (86)
Faculty rank	Instructor	2 (7)
	Assistant Professor	11 (38)
	Associate Professor	9 (31)
	Professor	7 (24)
Gender	Men	14 (48)
	Women	15 (52)

**Table 2:** Pseudonyms

Name	Race/ethnicity	Gender	Faculty rank
Ainsworth	African American	Male	Associate
Alameda	Latino	Male	Professor
Burnside	African American	Male	Associate
Bybee	Latino	Male	Professor
Foster	Native American	Male	Professor
Fremont	African American	Female	Clinical assistant
Grant	African American	Female	Assistant
Harold	African American	Female	Associate
Hawthorne	African American	Female	Associate
Hazelwood	African American	Female	Assistant
Morrison	African American	Female	Professor
Powell	African American	Female	Assistant
Prescott	African American	Male	Professor
Richmond	African American	Female	Assistant
Seward	African American	Male	Professor
Stark	African American	Female	Instructor

### Procedures

After receiving approval from our institutional review board, we contacted potential participants personally or via email. Following an informed consent process, authors DH, KFL, and AKB conducted 30–90-min interviews in person or by phone depending on participant location. Interviews were done in person (15%) and by telephone (85%). All interviews were audio recorded and transcribed verbatim. In the interviews, we asked participants to share their experiences as faculty of color in predominantly White schools of medicine. Interview topics included: 1) experiences of faculty of color over time; 2) relationships with others; 3) respect and value; 4) decision making and change; 5) job satisfaction; and 6) recruitment and retention of faculty and students of color. We also collected follow-up data to support development of the emerging theory with 20% of interviewees by repeat interview or email correspondence as described in the analysis section below.

### Grounded theory analysis

We conducted data analysis for all health professions schools data sets separately and then together. For every interview, members of the research team read transcripts and then open coded the transcript. Open coding means coding the data in ‘every way possible’ (35) We used the constant comparative method described by Glaser (35) and Bryan and Charmaz (28) to query the data, comparing data, concepts, and categories within and across interviews. Each team member engaged individually in this inductive and adductive process (33, 35). The team then compared, discussed, and analyzed interviews collectively to reach consensus on the analysis. Through focused coding, we selected and utilized frequent and significant codes developed through open coding to synthesize and analyze additional data (35). As data began saturating on core processes, we selectively coded for those categories. As categories and substantive codes began to approach saturation, we began developing theoretical codes. Theoretical codes ‘conceptualize how substantive codes may relate to each other as hypotheses’ to be integrated into the theory (35, p. 72). During theoretical sampling we expanded and completed the categories and tested relationships between categories to refine our theory. In studies using grounded theory, theoretical sampling can be accomplished using different techniques. We used three mechanisms: comparison across data sets, additional interviews, and revision and addition of interview questions.

To support development of the emerging theory, we conducted second interviews with selected participants to theoretically sample specific categories. This enabled us to further test and refine our model, and engage in member checking to test our interpretations and analytic categories with participants. We also shared our developing model and key categories with several participants for their feedback. Participants were selected for second interviews based on the richness of their first interviews, their ability to illuminate the properties, dimensions, and relevance of the developing categories, and their responsiveness and availability for follow-up interviews. We memoed throughout the analytic process, assisting theoretical development and promoting a more complex understanding of the experiences of faculty of color.

### Evaluation criteria

Criteria proposed by Charmaz for grounded theory studies in social justice inquiry include credibility, originality, resonance, and usefulness (29, 31). Combined with validity criteria for qualitative research from Whittemore et al., these serve as useful evaluation criteria (36).

Peer review and debriefing, member checks, analyst reflexivity, and rich thick descriptions enhanced the credibility of interpretation. We engaged in rigorous discussions and ongoing memoing, which helped us to maintain critical reflexivity and remain aware of and sensitive to the ways our own experiences influenced the data, as well as to our interpretation of the data and participants’ meanings. To evaluate theoretical credibility, we examined the data adequacy, category variation, and linkages between data, analysis, and presentation. We believe that the data presented in this article show that this grounded theory has theoretical and social significance and challenges current knowledge and practices. The resonance of the theory is reflected in categories that richly represent the experiences of the participants, while also accounting for multiple perspectives, complexity, and variation. The theory links participants’ actions with their implicit and explicit values as situated within the larger disciplinary, institutional, and sociocultural contexts. Supported by data, we have drawn linkages between the collective and individual lives of participants.

## Results

### Theory overview

**Fig. 1:**
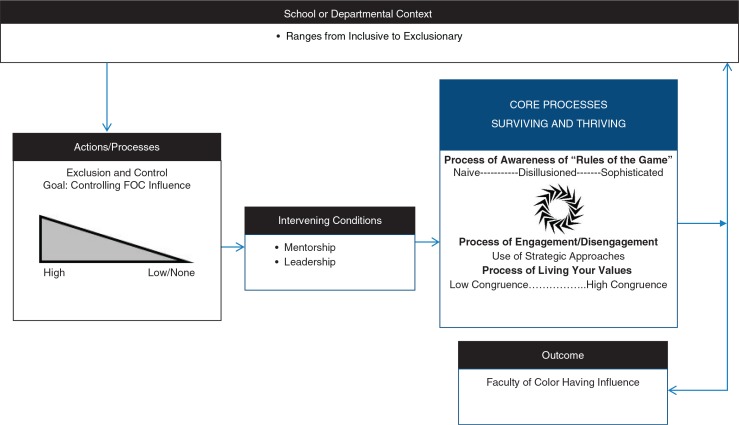
Representation of selected theoretical components.

Our theory, Faculty of Color in Health Professions Academe: Stories of Survival and Success, includes two key processes, exclusion and control and surviving and thriving. It also includes one outcome, having influence. Each of these happens in the school or department context that shapes faculty members’ risk of experiencing exclusion and control. In response to exclusion and control, faculty of color survive, thrive, or both depending on risk and protective conditions and context (Fig. 1). In our study, mentorship was the most frequently reported protective condition. We describe the entirely of our theory including the risk and protective conditions we identified elsewhere (37).

The final outcome was the ability of faculty of color to influence students, residents, patients, schools, and communities. Having influence gave faculty of color a sense of satisfaction because they were able to positively affect others, particularly other people of color.

### Patterns of exclusion and control

Exclusion and control are processes that restrict or limit faculty of color's influence on school cultures. Occurring in simultaneous, interrelated, and mutually reinforcing sub-processes, exclusion and control threaten faculty of color's career success while jeopardizing their well-being. Here, we provide examples of three exclusion sub-processes: invalidation of sense of self, othering, and unequal standards and access to resources.

#### Invalidation of sense of self

Invalidation of sense of self describes a series of ongoing personal assaults involving a failure to recognize faculty of color's individuality and professional knowledge. Instead, the faculty member is viewed as a member of a group first and an individual second, if at all. The following example comes from Dr. Stark, an African American woman who worked in a relatively inclusive context. She did not experience invalidation of sense of self in her environment:

I just felt like I fit here. And no one has treated me overly special because I'm Black, because … they're not walking on eggshells. They treat me as though I'm just me.

In contrast, Dr. Burnside, an African American man believed people's perceptions of individuality are generally secondary to skin color: ‘Ultimately … it's simply your skin color which will determine … how you're treated’.

Dr. Hawthorne, an African American woman also said:

Instead of seeing me as a … colleague, as an American, even as a woman, I think what they see me as first being Black and I think … with the face of being Black, they automatically assume that we are just completely different.

When skin color trumps individuality, a faculty member's credentials, knowledge, and judgment are viewed through the lens of group stereotypes. As Dr. Alameda, a Latino man noted: ‘What I hear from my colleagues who are African American is that people pass judgment on their ability before they open their mouth, because of the way they look’. The following example from Dr. Hawthorne supports this observation:

I've had other physicians also call … and question my diagnoses … and I think they have some apprehension based upon their typical imageries of people of African descent in this country so, therefore, to have someone in my position that looks like me is atypical … Unfortunately, the way that Blacks are personified in this country particularly … speaking of the Black females, it's been ornery and typically not very well educated.

Dr. Hawthorne also felt her clinical judgment was often ignored because of her race:

This is actually going on with the patient and they say, “No, that's not what's going on with the patient.” And two days later they say, “Oh, yeah. That's what was going on with the patient.” So it's … a matter of not respecting [my] judgment.

Invalidation resulted in faculty of color working extra hard to prove themselves to their colleagues, as Dr. Fremont explained:

I worked hard. I worked hard to dispel the myths that I was not capable …. Whenever you go to a place, you recognize that because you are not the same … whether it's a real issue or not, the perception you get is that you have to work extra hard.

These examples show that faculty of color, particularly those with darker skin, often experience invalidation of sense of self when, based on their race or ethnicity, they are sent negative messages about themselves and their abilities. We also found that particularly in medicine, these negative messages were also sometimes tied to gender. Specifically, for women of color gender intersects with race, creating a gendered, raced experience. This is consistent with the concept of intersectionality which denotes the ways in which race and gender interact to shape the multiple dimensions of women of color's experience (38). Our data suggests that for those faculty who experience negative racial, and for women negative racial and gendered messaging, over an extended period, there is a need to work to overcome lower self-confidence. Thus, they bear an emotional burden and face obstacles to success that majority faculty do not.

#### Othering

Othering is the second exclusion process we noted. Othering forms and sustains boundaries that maintain and police a group's character. Othering fosters a tendency to view one's own ethnic group as superior to all others and to assign meaning to other ethnicities using the dominant group as a standard. Thus, others are viewed as different and consequently outside of the dominant group. In our study, the primary means of othering was treating faculty of color as outsiders. As explained by Dr. Bybee, a Latino male participant:

There's not a day that goes by that I don't look at myself in the mirror and remind myself of who I am, meaning that, “Hey, remember, you're Latino …” And … I found myself feeling very marginalized, feeling very isolated, being very alone even though I'm in the Department of Family Medicine.

Being treated like an outsider may take the form of social exclusion, as experienced by Dr. Powell:

After we have conference or faculty meetings … they'll meet for lunch and go to the faculty dining hall. Well, I specifically know that I have just seen them at conference, and … nobody mentioned going to the faculty dining hall. So I just went anyway. And then it was just the awkward little … “Oh I guess we should invite her over to our table.” I mean, seriously … that's … just the way I took it, but that's the way I felt. Like I was intruding on their conversation.

More often, however, being treated like an outsider meant being excluded from meetings and decision-making as described in the following quote from Dr. Morrison:

We were – the two of us – supposed to be co-directors of our fellowship program. We were going to do the education and help with the fellowship program …. It turns out they started having meetings and I wasn't included. I wasn't invited; I wasn't included. I found out because we'd be in faculty meetings and they'd say, “Oh yeah, this is our plan …” And I'd say, “When did you meet?” I wasn't included; I wasn't invited. “Oh yeah, we forgot, but don't you worry, we took care of it. But we'll include you in the next one.” I was never included. And so that kind of thing kept going on …. Because it's about the race … It's not about anything else … the sheer fact that people still see me as an outsider.

As the above examples show, when faculty of color experience othering they are treated as members of an out-group, often encountering social isolation and exclusion from decision-making processes. Because URM faculty are already isolated by virtue of their numbers, the fact that many are othered by some majority faculty is very concerning. Likely this compounds their already existing isolation, contributing to feelings of loneliness and marginalization.

#### Unequal treatment

Unequal treatment is the third sub-process of exclusion we noted. It includes unequal performance standards and access to resources for faculty of color and majority members. Regarding performance standards, Dr. Prescott, an African American man, noted, ‘Faculty of color cannot afford to be average’. Dr. Morrison, an African American woman, experienced unequal treatment starting at the beginning of her career placing her at a significant disadvantage:

And going into medical school I had … a different set of views and values than other people … the traditional group that comes in …. And I did my fellowship in geriatrics … so when I finished up I was joining the faculty, but they were not very open to me … joining the faculty. In fact, the first year they listed me as staff, not faculty. They courted a woman who was also a friend of mine, and I had encouraged her, she was a year behind me, to do the fellowship. And she did, and they actively courted her, and I called them on it. I said, “Look at what you're doing for her and you didn't do any of that for me when I came in … you … put me down as staff and I was actually faculty.” Oh well, that's going to impact you [faculty of color] long-term.

Dr. Fremont, an African American woman, noted that she had difficulty getting placed on key committees:

How can people come behind you, they get put on … committees, they get positions, and you've been there and you don't get positions? … It doesn't make any sense to me. You … can't say that I'm not qualified …. Otherwise I wouldn't be there. So why should they come and get on committees and I've been there and I've had to ask and ask and ask and ask to be on a committee …. I've been hurt …. I felt like I contributed so much and I've not been appreciated for my contribution and I'm not sure why, other than the fact that I'm Black.

Whereas Dr. Prescott described unequal performance standards for faculty of color and majority faculty:

I think some of my majority colleagues cruise. Some … not all of them. Some of them cruise and that's tolerated. I know that won't be tolerated for me. I cannot be perceived as average. That won't go. So I got … awards last year …. I need that … just to stay in the game. There are other folks that I think cruised, they're not engaged in the full gamut of research, community service, patient care, and teaching. But they are de facto allowed to cruise because they are part of the ruling class. I am not allowed to cruise. And so I get frustrated when … I see myself being put in a position where I can't run [on] all accelerators.

Resource inequality often manifested as denial of protected time for faculty of color scholarship. Also, as experienced by Dr. Hazelwood, original commitments made to individuals were not consistently honored:

I was … promised one thing and then it didn't turn out that way …. [I've] been added a lot more clinical time since I've been here … I've been told things I felt were not fair to me. And … that if I didn't have a publication then my … research time wouldn't be supported … which I have found not to be true, equally true, for other members of the division.

Unequal treatment and access to resources impact faculty members’ ability to publish, write grants, and conduct research, placing faculty of color at a disadvantage when it comes to promotion and tenure. This covert inequality may contribute to the documented underrepresentation of URM at higher academic ranks within the professoriate and administration in US medical schools (21, 39). This inequality results in fewer senior faculty of color who can act as mentors, leaders, and role models for their junior peers, detrimentally affecting underrepresented groups.

To summarize, exclusion and control include simultaneous, interrelated, and mutually reinforcing sub-processes. Taken together, these patterns oppress and limit faculty of color in academic medicine and detract from the quality of academic environments for faculty of color and majority colleagues alike. Moreover, these patterns likely decrease retention of faculty of color, a critical issue in academic medicine.

### Surviving and thriving

We found that faculty of color responded to exclusion and control by surviving and thriving, the core social processes of our theory. Some of our interviewees appeared to stay firmly in survival mode, while others alternated between surviving and thriving or stayed primarily on the thriving end of the spectrum. Factors influencing whether a faculty member survived or thrived included level of mentorship and the severity of exclusion and control he or she faced.

Surviving and thriving comprises two interrelated sub-processes that occur against the backdrop of an awareness trajectory. The interrelated sub-processes are: the process of engagement and disengagement, and the process of living one's values.

The awareness trajectory denotes an understanding of the ‘rules of the game’ in a specific school or department. Some of our participants began their academic careers naïve to these rules and the impact racism may have on how they are enacted, specifically exclusion and control processes. When they discovered the existence of these processes, they became disillusioned. As they worked through their disillusionment, they gained valuable skills and moved into what we called the sophisticated stage. Sophisticated participants were those individuals who had developed a set of skills that enabled them to advance even in exclusionary contexts.

#### Strategic engagement and disengagement

Strategic engagement and disengagement were purposeful surviving and thriving strategies. Participants in the sophisticated stage had learned to engage intentionally with mentors, supportive colleagues, and trustworthy senior faculty within their schools and across institutions. They also collaborated with colleagues and students on personally meaningful scholarship and activities.

The most fortunate participants were engaged by others early in their careers. This set the stage for ongoing strategic engagement and thriving later. As explained by Dr. Foster, a Native American male faculty member who thrived in his role:

And I've been in that position for many years; I actually started here at this university. I was working on my doctorate at a neighboring institution and came here to work with a faculty member who was actually working with my tribe, and he was able to provide a minority supplement for me …. So, I've actually been here for 16 years, but … on the faculty for 10 years and so this is … really the only academic institution I've worked at. Although, I have … lots of colleagues at lots of different institutions.

As he progressed in his academic career, Dr. Foster went on to help other faculty of color. This underscores the critical importance of faculty of color success to subsequent generations:

I've had experiences with mentoring junior faculty … mentoring teens that are part of a diversity supplement … both at this institution and other institutions. I've had opportunities to mentor doctoral students …. I've had, I think, a pretty diverse experience … from junior faculty down to master (sic) level.

Faculty members who were not engaged early by mentors were sometimes able to seek out structured lectures and programs as a means of engaging with their academic environments. Dr. Fremont, an African American woman, recalls:

When I got here, of course, I wanted to be in academic medicine. So I knew that promotion was a goal for me. And I started … to get immersed in things that would allow me to move up on the ladder, so to speak. So I joined the woman's faculty organization. I went to presentations on … issues that affect women. I also tried to get into the scholars program to get some research going.

Engaging with professional organizations was also mentioned as a means of engagement with academic medicine overall. This is described in the following quote from Dr. Seward, an African American man:

Well … my mentor … sort of said, “You've got to come if you're going to be a player.” And I don't like that term, “player,” necessarily, but he said, “If you're going to be an influential person in – again, my cooperative group – you've got to come.” … So it wasn't too long after I actually got involved in a cooperative group that I was … a person they would look to ask questions.

Faculty of color also strategically engage by seeking supports. This involved seeking out sources of intellectual and emotional support, usually from minority colleagues. Dr. Grant, an African American woman, commented on the importance of processing feelings with an empathetic individual who understands what you are going through:

The isolation I think is real, especially during challenging times when … you need someone to … give you perspective about … how you feel …. It's not always dependent on ethnicity and gender but sometimes it can [be].

Participants commonly described feeling isolated and wanting supportive colleagues with whom they could talk things over. This need for an outlet prompted Dr. Harold, an African American woman, to create an informal club of minority faculty from different departments as a means of support:

Participant: If you have no solace, if you have no place of refuge, I think that that just takes your feeling of isolation and takes it to the Nth degree …. So, I have two outlets that I think are quite therapeutic. One is a professional organization and one was my little club group.

Interviewer: Mm-hmm. And both of those … involve faculty of color. Isn't that right?

Participant: Yes.

According to Dr. Harold, her ‘club’ was a place where she could talk through subtle instances of racism as well as everyday challenges:

… That whole concept of the microaggression …. What do you … do with all of that? … The majority of us don't sit around all day trying to be … that introspective and … trying to deal with all that on … a minute-to-minute basis. But it is real, it does exist, and it's something that's very difficult to quantify, and it's very difficult to convey to someone who has an otherwise experience. … And even if you could convey it, to have the security that in doing so, that you haven't otherwise ostracized yourself or given the impression that you are … weak, or you're unworthy in some other way, is a … heavy burden. So … where's the repository for all that? And I guess our club is a repository. Because … you tell it to people who, who get it, who know no further explanation is required.

Faculty of color also drew support from more formal gatherings organized by colleges and universities with diverse faculty, as reflected in this statement from Dr. Foster:

I think there's some solidarity between myself, as an American Indian faculty member, and our African American and Hispanic faculty. I think there's solidarity. I think because of our shared experience, because our numbers are relatively low, I think we really–for the most part–I think we realize if we work together we can make a difference, and so I'm part of that. I feel like I'm part of that experience where we're trying collectively to make a difference …. Twice a year we have a nice social gathering …. It's good to see when there's a new person that comes, it's great to see them and get to know them …. I would say there's a good solidarity among our faculty of color here.

As these participant quotes demonstrate, faculty of color develop emotional and intellectual supports to help them cope with the stress associated with exclusion and control and to help them understand and process these complex layered experiences.

The hallmark of strategic engagement is proactively seeking connections with mentors and colleagues to develop professional skills in addition to seeking emotional and intellectual supports from peers or even family members to protect well-being. Our data suggest that taking these steps often requires a strong awareness of exclusion and control processes combined with an awareness of individual needs.

#### Strategic disengagement

Although strategic engagement was often beneficial, disengagement was an effective coping strategy in certain circumstances. Strategic disengagement involved purposeful distancing from ones’ work environment or specific individuals. Participants had to learn the difference between strategic engagement and disengagement as well as when they were best implemented, and this knowledge came with sophistication. The following example illustrates how Dr. Harold used strategic disengagement to purposefully distance herself from invalidation of sense of self, which in this case was expressed through tokenism and demeaning comments.

Participant: Another example is some of the research that I was doing [with] … one of our fellows … looking at racial segregation. … I showed some of that research to my chair and other people and they're like, “Oh, this is happening stuff, you've got to present this and have this be … a grand rounds.” I don't really want to present it as a grand rounds. I don't really want that to be my … flag to carry, and my introduction to the rest of the faculty. They see that here I am – one of two African Americans – and I happen to be doing research on … disparities. … I'm interested in it, obviously, but I don't really want it to be my claim to fame, so to speak. … And so I've, I've shied away from it … and … avoid some of those conversations with people because I don't really want to hear what their reactions are going to be. Because if it's anything other than what I want to hear, then … that may color my further interactions with that person.

Interviewer: And … your hesitance to present that research is that because you're worried about being typecast? Or is it because you're worried how people are going to react and you know the work that you're going to have to do–emotionally and intellectually–to respond?

Participant: It is … equally distributed between my fear of being typecast and the diversity token. And … having to deal with the explanations and platitudes and further questions, etc., that are going to come about … Inevitably, I know the conversation's going to land up in a place that I'll probably disagree with … that person's thoughts, and I really don't want to know what their thoughts are. Because … I'm going to have to then carry that and figure out how to interact with this person on a regular day-to-day basis. So, not knowing is almost better.

In the preceding example, Dr. Harold is purposively avoiding diversity-related topics and conversations that she thinks will typecast her and lead to negative interactions with majority colleagues. She is purposefully choosing not to be exposed to what she believes will likely be her colleagues’ biased views to avoid personal distress. This is a very strategic form of avoidance for a participant who greatly values diversity. She is choosing her daily well-being, a healthy coping response. Because of the exclusion and control they routinely encounter, faculty of color often have to learn how to cope with negative ethnic or racial messages. Purposeful distancing is useful when used selectively and appropriately – a skill learned with sophistication.

#### Living one's values

Living one's values means being true to oneself. In our study, the majority of URM participants reported feeling responsible to help other people of color, whether they were students, residents, patients, or community members. This sense of responsibility was tied to a participant's personal values. Participants experienced value congruence when they enacted their values according to their sense of responsibility. However, juggling these perceived obligations with regular work responsibilities was a significant challenge. Learning how to modify and realistically align one's values and workload while achieving a modicum of career success and staying true to oneself came with sophistication.

In the following example, Dr. Morrison demonstrates values congruence. She explains where her own values came from and how they guide her actions in academia:

Because growing up in the South … none of us would have made it if we didn't have the support … of our own community …. We didn't have access to doctors. There were a couple of Black docs in that town that we could go to, but we all lived in the country. And people didn't have money. So our healthcare was literally practiced out of the church, and my grandmother and other older women, on Sunday, when we'd go to church … they would go from whatever vehicles people came in and – be it a horse and buggy or car or truck or whatever – and find out how families were, find out if somebody didn't come, who saw them. Because we didn't have any telephones, we didn't have any electricity. You rode past somebody; usually you'd stop to check on them. And so it was a big community thing. And so we looked out for each other and we supported each other … given what I know based on my history and the support I have received from those people who believed in me … I do believe I have a duty, a responsibility to help the next people come along. And to help even those people who … are not … there are other African Americans here, and they're, all of them are younger than I am …. Their commitment is to themselves and whatever their family is, their commitment is not to the community as I can see it. And when you ask them to do things–it's sort of, “Well, I'm so busy and this and that ….” I believe that because I have this awareness, that it is my responsibility to act on it. And maybe whatever those people are doing, they're doing what they're supposed to do or can do. But as hard as this is, it's something that I can do. So, that's why I stay–because I probably wouldn't feel right if I left.

Similarly, the following example from Dr. Ainsworth, an African American man, illustrates a high sense of responsibility and value congruence:

Okay, I may have to put up with a little bit of stuff, but I'm–I'm on a mission here, and I'm trying to get ahead, and I'm trying to help others get ahead. So we stay in not necessarily the best kind of environment. My environment isn't egregious. I don't come to work every day … with my head down, because I think someone's going to rate me or not treat me well. Indeed, it's closer to the other side, because I am a physician. But I recognize that … many people do not feel that I am their equal, and sometimes I'm tolerated, rather than appreciated. But nonetheless, this is the lot that I have for now, and I'm determined that the next generation of medical students, for them, it will be better, and for the next generation of students of color it will be better still.

In contrast, Dr. Harold was still trying to discover how to match her values with her workload and academic productivity:

It's not so much a microaggression, but it's the … minority tax, and being on every committee that has to deal with diversity in some way …. I'm on the women's committee, I'm on the diversity committee, and I'm on this committee of minority faculty affairs. I'm asked to be on panels all the dag-gone time. And you can't really. I guess you can, but my instinct is not to say no because they're important things, and geez, if we can't be there and represent, well then it's never gonna happen …. And if I don't sit on that student panel, then the students will never [have] someone who likes them, or will never aspire to be where I am, and then you have more issues and … the isolation is perpetuated. So I think many of us … feel a social obligation. To do it, when asked. I may not necessarily seek it out …. But then you also feel like, “Gosh, if I don't do it, who's going to do it?” Or, The next person that they get to do it probably won't do as good of a job as I tend to do …. Therefore, I should. And therefore, your calendar gets filled up with all these committee things and what not, which are otherwise not promotion generating activities …. And so you fall … into that trap. You're not productive, but you're busy … But in all the wrong ways.

Living one's values sustained faculty of color while sometimes simultaneously creating an added workload burden. Learning to stay true to oneself while negotiating the demands of the faculty role was a key to success for our participants. This finding highlights the importance of personal values in the careers of faculty of color in medicine. For those who feel compelled to engage in added mentoring and service activities living one's values can have significant workload implications. Learning to balance service with scholarship and to protect one's time are important strategies for success.

In summary, surviving and thriving includes two interrelated sub-processes whereby faculty of color cope with exclusion and control and promote their own well-being and success. The awareness trajectory provides a backdrop for these two sub-processes. Sophistication is the hallmark of strategic approaches to engagement and disengagement and living one's values. The use of sophisticated surviving and thriving strategies was an effective means of maximizing career success for the URM faculty in our sample.

### Outcome: faculty of color having influence

Despite the existence of exclusion and control, faculty of color in our study had a profound influence on students, residents, schools, patients, and communities. With influence came a sense of satisfaction, potentially a key variable in faculty retention. The following comment illustrates how important URM faculty are for student retention. Dr. Ainsworth's sense of satisfaction is evident:

They're [students] coming. But what I do tell them … this analogy–since it's Black History Month, I used this just the other day because I had attended … a little presentation–someone was doing a presentation of Harriet Tubman. And Harriet Tubman was talking about how she'd led people on the Underground Railroad to freedom and her train never went off the track and she never lost a person. I'm your male Harriet Tubman here at the school of medicine. I know how to get you through. I know how to get you to the Promised Land that you say you want. You want to become a physician. I know the pitfalls. I know the people to avoid. I know how much you need to study, and I will lead you if you come here and if you listen to me. Now, I understand Harriet Tubman would shoot you–if you didn't listen to her. Well, I'm doing none of that, but I–I tell the students–I almost guarantee them–if you follow my lead, I will get you the MD degree. These students have come here simply because of the support system that they perceive in me and my office. Because I've never lost a student …. That's what I tell them.

As Dr. Foster describes, participants also played critical roles as mentors to minority students:

We might have at any given time in our medical school, in our PA school … we might have three American Indian students across the medical center, and so … often times they will gravitate to me …. They would know me, they know my family …. So I feel a very special relationship with students in particular, because of that connection.

Dr. Harold offers mentorship to students interested in surgery and serves as a role model for African American women interested in becoming surgeons:

I just had a meeting with a student last week who's interested in surgery, it's an African American female, and she needs a wing, you know. And so … she's one of the people going to the professional meeting, and I avail myself. “Listen, I'm going, you and I will hang out; I'll introduce you to the people that you need to know. I'm going to help you along this journey …. We're just going to be proactive, we're going to make it happen and this is what I wished someone would do to me.” … I don't run out and seek out student interactions, but I get lots of students who come and make appointments with me because they–the Office of Multicultural Affairs–will funnel them my direction and so on and so forth. But I'm, I'm very frank when I speak to them. And this particular student, she wanted to know specifically, “Hey, I'm thinking of going into surgery and I'm an African American female, what do I need to look out for?” Which I thought was very nice that she … had the forethought … and the frankness to ask those questions. So, I don't really dance around topics and mince words …. If you ask me a direct question then I try to answer it, because … you're talking about people's lives and their livelihood. There's no room for false promises or telling someone that they're a good candidate, but otherwise they're not …. I'm not here to dash people's hopes or anything, but you have to be realistic with people, too …. I enjoy interacting with the students.

Dr. Richmond, an African American woman described the influence she had on majority residents, helping them to improve their cultural competence:

I think working with the residents, I can … help them begin to ask questions that maybe they wouldn't think of when they're trying to understand some of the problems that patients may have …. There are times when maybe I can raise issues and say …, “Think about what it would be like if you worried that your child might get hurt going to school because there's so much crime?” …. And those are things that … maybe they've never experienced. I can talk with them and say these are some of the things that my patients [experience]. I also think that it's a good thing both for the patients and the residents to actually see someone who looks like me is a physician, not just coming and saying, “Help me” …. I'm one of the people helping to solve the problems.

Indeed, patients of color were also frequently mentioned as an area where faculty felt they had important influence, as the following examples from Dr. Fremont and Dr. Richmond show:

We have diversity in the patient population. So I bring those unique abilities …. They say only the person wearing the shoe knows how it fits. So, since you're not of color, you don't know what it means to be in an environment …. And as a patient, you look for the best care (Dr. Fremont).

And the second example:

Dr Richmond: I have patients … “Oh, I'm so glad that I have you,” because they're coming and they want to talk about their experiences, their problems, and they feel very comfortable being able to talk to someone who looks like them and knows some of the things that they've been through. You know, they haven't just read about it in a book. That is a constant thing I see.

Interviewer: So does that give you a sense of satisfaction?

Dr. Richmond: It does. It really does. I feel like if I were in their place it would be awfully comfortable to have somebody who I didn't have to re-explain everything to …. There are a lot of cultural things that you can just sort of say … without having to explain it to someone.

Influence was also apparent at the school level in the form of diversity work and other forms of service, as reflected in this quote from Dr. Harold:

I tried to find out what resources were available for faculty, for minority faculty. They had lots of robust programs in place for medical students and pipeline programs and outreach and high school students and summer programs … but they didn't really have anything at all targeted to the faculty. So, I thought that that was kind of a shame. And I'd be recruited as a faculty member to come to these things to help support the students, but I didn't feel like anybody was supporting me. And I don't know if that was just … an oversight or just the assumption that well, you're self-sufficient because you've arrived …. But I never really felt like I arrived. I felt like I got a little guest pass and I needed to be able to change that and exchange it into a permanent … citizenship, so to speak. So because there was nothing there, I decided to try to create something and that was a big labor of love … just because we're so scattered. Anyway, we had this … mentoring list-serve for the medical students that I used as a basis to send out emails and try to see who among the faculty was interested in forming a group. We've since formed a committee on faculty affairs, we've actually now become a standing committee through the Dean's office, and are sanctioned by him, and actually have the budget, etc., but that's taken a very long time to get to.

Finally, as Dr. Morrison explains, participants also influenced members of their communities:

I get a lot of students who come in … telling me their stories, and some of the students can be high school students because I'm trying to mentor them. They will call me to mentor them because I'm the African American in the health sciences that they see and will spend time talking with them. And so, because I do a lot in the community … people know me for the most part, and they find me to be approachable, so … their kids will want to come and talk.

To summarize, URM faculty in our sample had widespread influence despite the numerous barriers they encountered in academic medicine. They played critical roles as faculty members, mentors, and teachers, improving the educational experiences of all students and residents and the quality of patient care for diverse populations. This finding documents the vital importance of their success for successive generations of faculty and, by extension, the quality and accessibility of US health care.

## Conclusions and implications

Our findings are consistent with the existing literature in academic medicine and in higher education generally related to the importance of faculty of color (faculty of color having influence) (1, 6, 13, 14, 19), racism in academe (exclusion and control processes) (6–11, 16, 17, 19, 20, 40), and survival and success strategies for faculty of color (surviving and thriving) (12, 23, 24, 26, 27). What our theory offers is a framework to fit these findings together (see Fig. 1) while expanding the limited evidence base specific to medicine. Exclusion and control processes are conceptualized as interrelated and co-occurring with specific effects. The presence of exclusion and control in academic medicine likely explains in part the need for intensive faculty development and support programs targeting URM faculty (15). Invalidation of sense of self occurs over an extended period often resulting in lowered self-confidence among faculty of color. This finding suggests that concerted efforts should be made to counteract these negative messages by providing faculty of color with positive feedback about their abilities and their work. In addition, faculty of color themselves should be aware of the impact negative messages may have on their psyche and the need to counteract negative effects with positive self-talk and make a concerted effort to seek positive support from others. Othering results in social isolation and exclusion from decision-making processes. This finding highlights the importance of having a critical mass of faculty of color to minimize feelings of isolation. Because othering of faculty of color may occur as a result of unconscious bias (41), this finding also suggests that White faculty members should make a concerted and conscious effort to be inclusive in their interactions with faculty of color to counteract this process. Unequal treatment and access to resources places faculty of color at a disadvantage when it comes to promotion and tenure, likely explaining inequalities in representation at the higher ranks (21, 39). This finding highlights the need for organized initiatives that can help faculty of color, and in particular faculty from groups that are underrepresented at the higher ranks, to successfully work toward and attain promotion and tenure in schools of medicine to redress this inequity.

Surviving and thriving is conceptualized as processes occurring on a continuum with faculty moving back and forth between the two ends of the spectrum. As faculty of color learn the rules of the academic game and to effectively manage exclusion and control processes in academe, they gain sophistication. This is associated with awareness of how the rules of the academic game interact with exclusion control processes, and of one's personal values. These skills include learning to engage and disengage strategically and living one's values. The hallmark of strategic engagement is proactively seeking connections with mentors and colleagues to develop professional skills in addition to seeking emotional and intellectual supports from peers or even family members to protect well-being. These findings underscore the importance of taking a thoughtful and deliberate approach to one's career and need for good mentoring to engender these skills. Training for mentors related to the specific issues faced by faculty of color in academic medicine as well as continued development of mentoring programs targeting URM faculty are recommended. Living one's values sustained faculty of color at the same time that it sometimes created an added workload burden. Learning to stay true to oneself while negotiating the demands of the faculty role was key to success for our participants. This finding highlights the importance of personal values in the careers of faculty of color in medicine. It also suggests that medical schools should recognize their contributions including mentoring junior peers and students of color and diversity-related service in the forms of positive messaging, workload credit, and promotion and tenure service criteria.

The outcome of surviving and thriving is faculty of color having influence. Our participants’ influence on their students, residents, patients, schools, and communities was profound. These findings support the importance of promoting not only URM recruitment and retention, but also faculty success. Each faculty member is instrumental to the survival and success of others. Loss of one URM faculty member likely means loss of benefits to many students, residents, patients, and community members as well as the school environment. Thus, we encourage schools of medicine to consider making URM faculty support a high priority and indeed, part of their core missions. With intentional, consistent, and thoughtful effort, URM faculty can succeed in academic medicine. The importance of efforts to the future of medicine cannot be overstated.

## Suggestions for future research

Our data suggest that the contributions of all faculty of color are substantial and that there are significant commonalities in their experiences. However, qualitative differences in experience according to racial and ethnic group, gender, skin color, and immigrant status also exist. Future qualitative research focused on specific intersectional groupings to further explore the complexities of the experiences in these various social locations and advance the knowledge base in academic medicine is needed.

## References

[1] American Association of Medical Colleges (2009). Striving toward excellence: faculty diversity in medical education.

[2] U.S. Department of Health and Human Services Healthy people 2020 objective topic areas 2014.

[3] Institute of Medicine (2002). Unequal treatment: confronting racial and ethnic disparities in health care.

[4] Sullivan Commission on Diversity in the Health Care Workforce (2004). Missing persons: minorities in the health professions.

[5] Cook M, Irby D, O'Brien B, Schulman L (2010). Educating physicians.

[6] Turner CSV, González JC, Wood JL (2008). Faculty of color in academe: what 20 years of literature tells us. J Divers High Educ.

[7] Carr P, Paplepu A, Szalacha L, Caswell C, Inui T (2007). Flying below the radar: a qualitative study of minority experience and management if discrimination in academic medicine. Med Educ.

[8] Cora-Bramble D, Zhang K, Castillo-Page L (2010). Minority faculty members’ resilience and academic productivity: are they related?. Acad Med.

[9] Mahoney M, Wilson E, Odom K, Flowers L, Adler S (2008). Minority faculty voices on diversity in academic medicine. Acad Med.

[10] Pololi L, Cooper LA, Carr P (2005). Race, disadvantage and faculty experiences in academic medicine. J Gen Intern Med.

[11] Price EG, Gozu A, Kern DE, Powe NR, Wand GS, Golden S (2005). The role of cultural diversity climate in recruitment, promotion, and retention of faculty in academic medicine. J Gen Intern Med.

[12] Alexander R, Moore SE (2008). Introduction to African Americans: benefits and challenges of working in predominantly White institutions: strategies for thriving. J Afr Am St.

[13] Jayakumar UM, Howard TC, Allen WR, Han JC (2009). Racial privilege in the professoriate: an exploration of campus climate, retention, and satisfaction. J High Educ.

[14] Hassouneh D, Lutz K (2013). Faculty of color having influence in schools of nursing. Nurs Outlook.

[15] American Association of Medical Colleges (2012). Diversity in medical education facts and figures 2012.

[16] Cartwright BY, Washington RD, McConnell RL (2009). Examining racial microaggressions in rehabilitation counselor education. Rehabil Educ.

[17] Hassouneh D, Akeroyd J, Lutz K, Beckett A (2012). Exclusion and control: patterns aimed at limiting the influence of faculty of color. J Nurs Educ.

[18] Lin A, Kubota R, Motha S, Wang W, Wong S, Guofang L, Gulbahar BH (2006). Theorizing experiences of Asian women faculty in second- and foreign-language teacher education. “Strangers” in the academy.

[19] Stanley CA, Stanley CA (2006). Overview of the literature. Faculty of color – teaching in predominantly White colleges and universities.

[20] Hodge SR, Wiggins DK (2010). The African American experience in physical education and kinesiology: plight, pitfalls, and possibilities. Quest.

[21] Nunez-Smith M, Ciarleglio M, Sandoval-Schaefer T, Elumn J, Castillo-Page L, Peduzzi P (2012). Institutional variation in promotion of racial/ethnic minority faculty at US medical schools. Am J Public Health.

[22] Allen WR, Epps EG, Guillory EA, Suh SA, Bonous-Hammath M, Stassen M, Smith WA, Altbach PG, Lomotey K (2002). Outsiders within: race, gender, and faculty status in U.S. higher education. The racial crisis in American higher education.

[23] Lutz K, Hassouneh D, Akeroyd J, Beckett A (2013). Balancing survival and resistance: experiences of faculty of color in predominantly Euro-American schools of nursing. J Divers High Educ.

[24] Stanley CA, Stanley CA (2006). Summary and key recommendations for the recruitment and retention of faculty of color. Faculty of color – teaching in predominantly White colleges and universities.

[25] Turner CS, Myers SL, Creswell JW (1999). Exploring underrepresentation: the case of faculty of color in the midwest. J High Educ.

[26] Adams SG, Stanley CA (2006). Succeeding in the face of doubt. Faculty of color – teaching in predominantly White colleges and universities.

[27] Alfred MV, Mabokela RO, Green AL (2001). Success in the ivory tower – lessons learned from Black tenured female faculty at a major research university. Sisters of the academy – emergent Black women scholars in higher education.

[28] Bryant A, Charmaz K, Bryant A, Charmaz K (2007). Introduction to grounded theory research: methods and practices. The Sage handbook of grounded theory.

[29] Charmaz K, Denzin N, Lincoln Y (2005). Grounded theory in the 21st century: applications for advancing social justice studies. The handbook of qualitative research.

[30] Denzin N, Bryant A, Charmaz K (2007). Grounded theory and the politics of interpretation. The Sage handbook of grounded theory.

[31] Charmaz K, Denzin N, Lincoln Y (2011). Grounded theory methods in social justice research. The Sage handbook of qualitative research.

[32] Kincheloe JL, McLaren P, Steinberg SR, Denzin N, Lincoln Y (2011). Critical pedagogy, and qualitative research: moving to the bricolage. The Sage handbook of qualitative research.

[33] Glaser B, Strauss A (1967). The discovery of grounded theory: strategies for qualitative research.

[34] Harding S, Alcoff L, Potter E (1993). Rethinking standpoint epistemology. Feminist epistemologies.

[35] Glaser B (1978). Theoretical sensitivity.

[36] Whittemore R, Chase S, Mandle C (2001). Validity in qualitative research. Qual Health Res.

[37] Hassouneh D, Lutz KF (in progress). Faculty of Color in the Health Professions: Stories of Survival and Success.

[38] Crenshaw KW, Crenshaw K, Gotanda N, Peller G, Thomas K (1995). Mapping the margins: intersectionality, identity politics, and violence against women of color. Critical race theory – critical writings that formed the movement.

[39] Fang D, Moy E, Colburn L, Hurley J (2000). Racial and ethnic disparities in faculty promotion in academic medicine. JAMA.

[40] Harley DA (2008). Maids of academe: African American women faculty at predominantly White institutions. J Afr Am St.

[41] Hassouneh D (2013). Unconscious racist bias: barrier to a diverse nursing faculty (editorial). Journal of Nursing Education.

